# Parotitis Following Typhoid Fever

**Published:** 1903-02

**Authors:** E. Bates Block

**Affiliations:** Lecturer on Nervous Diseases, Atlanta College of Physicians and Surgeons; Visiting Physician to the Presbyterian Hospital of Atlanta and the Tabernacle Home and Infirmary


ATLANTA

### Journal-Record of Medicine.

Successor to Atlanta Medical and Surgical Journal, Established 1855, and Southern Medical Record, Established 1870.

VOL. IV. FEBRUARY, 1903. No. 11.

BERNARD WOLFF, M.D., EDITOR, Nos. 319-20 Prudential.

Published Monthly.

M. B. HUTCHINS, M.D., BUSINESS MANAGER, No. 64 Marietta St.

### ORIGINAL COMMUNICATIONS.

### PAROTITIS FOLLOWING TYPHOID FEVER.*

BY E. BATES BLOCK, M.D.,

Lecturer on Nervous Diseases, Atlanta College of Physicians and Surgeons; Visiting Physician to the Presbyterian Hospital of Atlanta and the Tabernacle Home and Infirmary.

The case of parotitis following typhoid fever which I am about to report was under the care of my colleague, Dr. Hull, and myself at the Presbyterian Hospital last fall. Although a very rare complication or sequel of typhoid fever, it may serve as a starting point for a consideration of some very interesting data which have accumulated in the last decade concerning the distribution of the typhoid bacillus in the body, its pyogenic character and association with other microbe organisms, and parotitis following other diseases.

In a series of three hundred and eighty-nine cases reported by Osler, treated in the Johns Hopkins Hospital, parotitis occurred

* Read before the Atlanta Society of Medicine, and the Medical Association of Georgia.

in only six cases, making a percentage of 1.54. In one of these cases there was double parotitis, in two only the right parotid was affected, and in three only the left. Two of these cases subsided without operation. In two of the cases reported in detail, one by Flexner, the temperature fell before the parotitis was noticed. In Flexner's case the parotitis was not noticed until the day of death, while in Osler's it occurred on the seventeenth day of the disease. In the following case the parotitis developed during convalescence, on the forty-seventh day of the disease.

J. W. J., male, aged thirty-four, was admitted to the Presbyterian hospital of Atlanta on 26th of September, 1901—on the sixth day of his illness, complaining of weakness, dizziness, headache, and constipation, with soreness in the abdomen. Patient had had lues in 1886 and was treated for three years. In 1899 had synovitis of knee-joint. Otherwise his past history was negative. His temperature on admission was 104° F.; pulse 118. The face was flushed. There were no rose spots. There was general abdominal tenderness, but no gurgling. The spleen was not palpable.

The urine showed a specific gravity of 1019—acid reaction—no albumin—no sugar.

On 30th of September rose spots were noted on the abdomen. On 2d of October a bed-sore developed over the sacrum, about the size of a dollar. Both cheeks were flushed. The tongue showed a heavy white coat. The spleen was distinctly palpable.

During his illness the temperature ranged between 102° and 103.3°, and fell by lysis, reaching a steady normal temperature on 19th of October.

On the 22d of October the tongue still had a light coat; the spleen still palpable; temperature normal; no abdominal tenderness or pain.

On 4th of November patient complained of pain at the angle of jaw on left side. There was some swelling and slight redness in region of left parotid.

On 5th November the pain had greatly increased. There was considerable swelling in the region of the left parotid gland, which presented a hard, resisting mass, which was very painful upon pressure.

The temperature rose to-day to 101.3°, after having been normal or subnormal (usually) for sixteen days. The temperature then fluctuated between subnormal and 100° F. until the 10th of November, after which it remained practically normal, with the exception of five rises to 99° F. between the 10th day of November and the time of discharge from hospital on 28th November. The rise of temperature accompanying the swelling of the parotid was on the forty-eighth day of the disease, one day after the swelling was first noticed. The swelling extended from about one-half inch above the lobe of the ear to about two inches below the angle of the jaw, and covered the angle. The swelling was treated by hot fomentations, and on the 6th of November there was very slight pain, although there was no change in the size of the mass. On 7th November the pain had disappeared and the swelling much smaller, but on the 8th pain returned and the gland again became swollen and red, with a rise of temperature to 100° F. Hot fomentations were repeated and the swelling quickly subsided without evidence of suppuration.

The patient was given cold sponges for his fever, and aside from a little calomel and capsules of salol and podophyllin quite early in his illness received no other treatment. From the time of admission he had from three to eight loose stools a day. Diarrhea ceased about the 15th of October, before the temperature reached normal, after which there was constipation, the bowels moving only after enemata.

Other cases of parotitis following typhoid fever are reported by Osler, Flexner, Atkinson, Paget, and Murchison. According to Paget, out of two thousand typhoid patients in the London Fever Hospital during the years 1870–1885, thirteen had parotitis. The celebrated Henle reported an observation on himself in which the parotitis was followed by unilateral sweating on the affected side. Berard and Pokroffsky reported parotitis each following a febrile disease, and Parfianovitch and Wilde reported cases following typhus fever.

The responsibility of the typhoid bacillus for inflammatory processes, even pus formation, is well shown by the following list of observations taken mainly from the reports of Duflocq, Flexner,

Ohlmacher, and Parsons. The typhoid bacillus has been found in: *Thyroiditis* by Dupraz, Spirig (associated with Staphylococcus Albus), Colzi, Honl, Jeansalme, and Golgi; *Orchitis* by Tavel, and Salles and Barjon; *Epididymitis* by Girode and Gilbert; *Ovarian Abscess* by Verth; *Abscess of the Kidneys* by Gallois and Flexner; *Localized Peritonitis* by Fraenkel and Lehman; *Meningitis* by Fernet, Stuehlen, Daddi, Tictine, Ohlmacher, Silva, Honl, Vincent, Hintze, Mensi and Carbone, Kuhnau and Kamen; *Pleurisy* by Valentini, Loriga and Pensuti, Ferner, and Charrin and Roger; *Abscess of the Liver* by Lannois and Lyonnet; *Purulent Cholecystitis* by Gilbert and Girode, and Chiari; *Cutaneous or Subcutaneous Abscesses* by Chantenesse and Widal, Raymond, and Daddi; *Acute Endocarditis* by Carbone, Vincent, and Girode; *Abscess of the Spleen and Mesenteric Glands* by Roux and Vinay, Lehman and Flexner; *In Suppurative Processes of Bones or Periosteum* by Valentini, Ebermaier, Golgi, Orlow, Pean and Cornil (associated with other organisms), Achalme, Broca, Achard, Sultan, Buschke, Bruni, Parsons, Orloff, Colzi, Melchoir, Dupraz, and Catrin; *In Suppurative Costal Chondritis* by Helferich, Widal, Achard, and Broca. It may be mentioned here that Quincke found the typhoid bacillus almost constantly present in the bone marrow of typhoid patients, without any lesions being presented, and its practically constant occurrence in the spleen and mesenteric and lymph glands is well known. Further suppuration has been produced experimentally in animals by injection of typhoid bacilli by Mya and Belfanti, Dmochowski and Janowsky, and Block.

I have not been able to find in the literature a single case in which the typhoid bacillus was found in parotitis. In Flexner's case he obtained a pure growth of streptococcus pyogenes, and in the case reported by Atkinson I took cultures twice from the suppurating parotid, and each time obtained a pure growth of staphylococcus pyogenes aureus. In spite of this fact we are aware that the typhoid bacillus is at some time in the disease widely disseminated throughout the body, for it has been found in the blood during life by Thiemich, Ettlinger, Stern, Block, Kuhman, and Singer; and from the rose spots in thirteen out of thirty-eight cases by Neuhauss, Rutimeyer, and Thiemich; and it has been found in the urine in thirty-nine out of one hundred and fifty-three cases by Wyssochowitch, Hueppe, Seitz, Konjajeff, Neumann, and Karlinski.

It is therefore not improbable that the typhoid bacillus may yet be found in the parotid inflammation following typhoid fever. As has already been pointed out, the cases which have been examined bacteriologically have shown either streptococci or staphylococci, and in a previous paper I have called attention to the importance of the association of other bacteria with the typhoid infection. The chief danger seems to lie in the association of typhoid with streptococcus infection. In forty-one abscesses in typhoid cases examined by Vincent the staphylococcus pyogenes aureus was present thirty-two times, all of these patients recovering. In eight cases streptococcus alone, or associated with the bacillus typhosus, was met with, and of these five died. In the condition of lowered vitality and resistance which exists in typhoid fever the patient seems very susceptible to infection by other micro-organisms which are often responsible for the complications, though not always, as previously shown.

Parotitis does not follow typhoid fever exclusively; it may indeed occur in any of the exanthemata, septicemia, or dysentery, and Paget has collected cases of parotitis following injury and diseases of the peritoneum, generative organs, and abdominal viscera. Among these cases the parotitis followed the use of the catheter in a case with enlarged prostate and irritable bladder; the use of a sound in a similar case; cystitis; peritonitis (?) and septicemia after delivery; induced abortion; peritonitis after delivery; retarded convalescence after delivery; pyemia after delivery; after delivery and puerperal mania; traumatic peritonitis; peritonitis; peritonitis from perforation of the appendix; peritonitis with pyelonephritis; pyemia from bullet wounds of lumbar spine (President Garfield); malignant disease of lumbar spine with subperitoneal abscess above the symphysis pubis; peritonitis with perforation of duodenal ulcer; symptoms of gastric ulcer; gastrotomy; gastrostomy; enterostomy; cancer of the sigmoid flexure with intestinal obstruction; cancer of sigmoid flexure, abdominal section and colotomy; herniotomy; removal of omentum and pyemia; acute jaundice and erysipelas; ovariotomy and peritonitis; ovariotomy and perimetritis; ovariotomy; ovariotomy and abortion; pelvic cellulitis; pelvic abscess and cancer of uterus; operation for lacerated cervix; penetrating wound of abdomen; operation for vesicovaginal fistula; following Tait's operation; after hysterectomy; incision of the cervix with pregnancy; from a pessary; a blow on the testicle.

Goodell, in a paper read before the American Gynecological Society, reported five cases of inflammation of the parotid glands following operations on the female genital organs, and ten were added by other members. These cases were mentioned by Paget, who says further: "The cases of parotitis following injury, not of the generative organs, but of the abdominal viscera and peritoneum, may perhaps show that the relation of mumps to orchitis is not due to any connection between the salivary glands and sexual glands, but to some wider connection between the glands at the beginning of the alimentary canal and the membrane which is reflected over the rest of it and over the testicles and the female generative organs." "On the other hand, if the case of parotitis after circumcision, quoted above, was more than a mere coincidence, and if the few published cases of inflammation of the breast and labia in mumps were more than a coincidence, then there may be some special bond between the salivary organs and sexual organs." In a later paper Paget says: "It is probable, (1) that the parotid is related to the peritoneum; (2) that it is also related to the generative organs; (3) that an abdominal or pelvic lesion may be followed by parotitis without pyemia." Taylor took a very different view of the subject, and seemed decidedly in favor of the septic theory, and it must be remembered that this complication is really a very rare one, and is by no means limited to lesions of the abdominal or generative organs. Thus Paget, in an article on "The Distribution of Pyemic Abscesses," relates a case of an abscess on the back of the left hand and double parotitis, and Greenwood records a case of right parotitis complicating acute endocarditis following rheumatism. Nevertheless, there are cases which it would be impossible to understand unless we believe that there exists some special relation between these distant parts. One of the most remarkable cases on record is reported by Peter. In a case of amenorrhea there was left parotitis at the menstrual periods, alternating occasionally with swelling of the labia on menstruation. In May there was parotitis, in June parotitis, in July swelling of the labia, in August parotitis, in September parotitis, in October menstruation, no swelling of parotid or labia, in November swelling of the labia. And Harkin reports a case in a woman forty-three years old who had enlargement of the parotid gland in six successive pregnancies, this being one of the first indications of her gravid condition. Whether there exists any relation between the parotid in mumps and the ovary has even been called in question on account of its rarity, although its relation to orchitis is generally admited. There seems, however, to be some such relation. Cribb reports two cases of mumps in young ladies, aged twenty and twenty-two, complicated by painful and swollen ovaries. And Moricke observed five cases of parotitis following operation, out of two hundred ovariotomies, and none after any other operation. Four of these cases suppurated. Dr. Davis informs me that he has seen two such cases after ovariotomy. Paget collected twenty-seven cases of parotitis following ovariotomy, occurring usually in the first or second week following the operation. (These include most of those mentioned above.)

Paget collected 101 cases of parotitis following injury or disease of the abdomen or pelvis. "Of the 101 cases 10 were due to injury or disease of the urinary tract, 18 were due to injury or disease of the alimentary tract, and 23 were due to injury or disease of the abdominal wall, the peritoneum or the pelvic cellular tissues. The remaining 50 were due to injury or disease or temporary derangement of the generative organs. By 'temporary derangement' I mean slight injuries or natural processes, a slight blow on the testicle, the introduction of a pessary, menstruation, and pregnancy." "This form of parotitis after abdominal or pelvic injury or disease is not, as a rule, accompanied by signs of septicemia or pyemia. Thus out of 101 cases there are only 15 where mention is made of 'septic symptoms' or 'septicemia' or 'pyemia.' "Parotitis, when it follows injury or disease of the abdomen or pelvis is in 93 per cent. of the cases a solitary event, unaccompanied by any other lesion like itself." "Thirty-seven cases died, but of these 3 were over eighty years old, 3 had internal cancer, 2 had perforation of the bowels, 2 had strangulated hernia, 7 had undergone severe operations involving abdominal section, and 13 died of septicemia or pyemia. One died from infantile syphilis, 1 from marasmus, and 1 from heart disease." "Out of 78 cases, which give information on this point, 45 suppurated, and 33 were resolved without suppuration. Out of the 45 that suppurated 24 died, but out of the 33 which were resolved without suppuration only 1 died, and she died of cancer. But the suppuration of the parotid was not the cause of death in the 24 who died; they died of the primary injury or disease. They did not die because their parotitis went on to suppuration, but their parotitis went on to suppuration because they were going to die."

Paget advances a number of reasons for believing that the parotitis in this class of cases is due to reflex nerve influence. If this be true, what path does the reflex take; does it act on the same side, or does it cross over to the opposite side? Unfortunately the side of the lesion has not been specified in many of the cases, but I have been able to find a few. Goodell reports a case in which there was a congested and tender left ovary; the left parotid gland does not secrete during menstruation, and then the mouth and fauces on that side are dry and painful (Paget). Paget cites a case of ovariotomy, pus in the left ovary, pyemia, left parotitis. In both of these cases the lesion of the parotid was on the same side as the lesion of the ovary, but in a case of removal of a papillomatous tumor of the right ovary there was swelling of both parotids. In 3 cases the parotitis developed on the side opposite to the other lesion. A retroperitoneal tumor was removed with the left kidney to which it was adherent, and right parotitis followed (Hulke's case cited by Paget.) Osler reported a case of typhoid fever complicated by parotitis on the left side with ovarian hemorrhage on the right side. Debout d'Estrees reported an interesting case of gouty parotitis disappearing on invasion of the knee of the opposite side. The other parotid and other knee subsequently became affected. From such limited data it is impossible to say whether the reflex nerve influence, if it exists, crosses or acts on the same side. It may be worthy of note that in a case of removal of both the ovaries, of which the left was hypertrophied and cystic, there was first right parotitis and later left parotitis. Also in typhoid fever either one or the other, or both, parotids may be affected. It is very difficult to form a conception of suppuration being independent of bacterial agencies. Nevertheless, Paget seems to attribute the swelling to reflex nervous influence, and indeed it is curious that other neighboring parts seem to bear a relation to the generative organs, as shown by the swelling of the nasal mucous membrane in certain menstrual troubles. Mackenzie, who has written quite an extensive article on the relation of the nasal mucous membrane to the generative organs, quotes Fliess's observation that cocainization of one nostril causes disappearance of the pain on the other side of the body in dysmenorrhea.

One point that Paget brings in support of the reflex nervous theory, viz., that diseases of the thoracic viscera cause inequality of the pupils, permits of quite a different explanation. It is usually in diseases of the apex of the lung, or aneurysm, involving the cervical sympathetics, that inequality of the pupils is observed. I was not aware that abdominal or pelvic lesions ever caused inequality of the pupils or disturbances in the reactions, and I would be very grateful for any information on this subject. However, it is well known that there exists some nervous reflex relation between the generative organs, the breast and the nose, and it lies nearer to assume that such a relation may influence the parotid gland. While such influence may contribute to the swelling of the parotid, it must, if it exists, play only a minor part in typhoid fever, for it is too well known that the parotid often goes on to suppuration, which has, in a few cases at least, been demonstrated to be due to well known pyogenic organisms (Atkinson, Flexner, Rosendach). It seems, however, that an infective theory would not hold true for all cases in which there is simple swelling of the parotid, without suppuration, following diseases of the viscera covered by peritoneum and diseases or injury to the generative organs. It is essential, in order to establish a reflex nerve theory, that all bacterial causes should be excluded by careful bacteriological examination in a number of cases. It is quite possible, however, that a reflex nerve influence may render the parotid more susceptible to infection. The subject needs closer and more careful study.

REFERENCES.

1. Atkinson. *Johns Hopkins Hospital Bulletin*, 1897.

2. Berard. Quoted by Farwick; Einseitige Hyperhidrosis. *Inaug. Diss., Mainz.*, 1881.

3. Block. *Johns Hopkins Hospital Bulletin*, 1897.

4. Cribb. *Lancet*, 1886. Jan., p. 227.

5. Debout d'Estrees. On Gouty Parotitis and Gouty Orchitis. *Brit. Med. Journ.*, March, 1887, p. 569.

6. Duflocq; Lecons sur les Bacteries Pathogenes. Paris, 1897.

7. Ettlinger. Wurtz; Precis de Bacteriologic Clinique.

8. Flexner. *Johns Hopkins Hospital Reports*, Vol. 5, p. 343 *et seq.*

9. Goodell. Abstract in *Medical Times and Gazette*, 1886 (cited by Paget), and *American Journal of Obstetrics*, 1886 or '7.

10. Greenwood. Parotitis. *Brit. Med. Journ.*, March, 1887, p. 676.

11. Harkin. The connection between the parotid glands and the generative organs. *Lancet*, Feb., 1886, p. 374.

12. Henle. Quoted by Farwick. Ueber Einseitige Hyperhidrosis. *Inaug. Diss., Mainz.*, 1881.

13. Kuhman. Quoted in *American Journal of Medical Sciences*, 1897.

14. Mackenzie. *Johns Hopkins Hospital Bulletin*, 1898.

15. Moricke. *Med. Times and Gazette*, 1881, Vol. 2, p. 270 (cited by Paget), and *Zeitschr. f. Geburtshulfe u. Gynaec.*, Vol. 5, p. 348.

16. Murchison. Allbutt's System of Medicine, Vol. 1, p. 820.

17. Ohlmacher. *Journal of the American Medical Association*, 1897.

18. Ohlmacher. *Cleveland Medical Gazette*, 1897.

19. Osler. *Johns Hopkins Hospital Reports*, Vols. 4 and 5.

20. Paget, Stephen. The distribution of pyemic abscesses. *Lancet*, 1886, July, p. 203.

21. Paget, S. Parotitis after injury or disease of the abdomen or pelvis. *Brit. Med. Journ.*, March, 1887, p. 613.

22. Paget, S. Secondary inflammation of the parotid. *Lancet*, April, 1886, p. 732.

23. Paget, S. The relation of the parotid to the generative organs. *Lancet*, Jan., 1886, p. 86.

24. Parfianovitch. *Brit. Med. Journ.*, 1887, p. 78.

25. Parsons. *Johns Hopkins Hospital Reports*, Vol. 5.

26. Peter. *Gaz. des Hop.*, 1868, p. 146 (cited by Paget).

27. Pokroffsky. Ein Fall von Hyperhidrosis Unilateralis. *Berliner Klin. Wochenschrift*, 1875, p. 164.

28. Singer. *Wiener klin. Woch.*, 1896.

29. Stern. *Centralbl. f. innere Med.*, 1896, p. 1249.

30. Taylor, F. T. The relation of the parotid to the generative organ. *Lancet*, Jan., 1886, p. 130.

31. Thiemich. *Baumgartens Jahresbericht*, Vol. 10, 1894, p. 266.

32. Vincent. *Annales d l'Inst. Pasteur*, 1883.

33. Wilde. *Brit. Med. Journ.* 1887, p. 675.

